# Research on the impact of delayed retirement on the subjective wellbeing of older adults

**DOI:** 10.3389/fpubh.2025.1530613

**Published:** 2025-06-10

**Authors:** Guo-feng Guan, Lu Wang, Dong-fen Ma, Pan-shi Luo

**Affiliations:** ^1^School of Economics, Xinjiang College of Science and Technology, Korla, China; ^2^School of Finance, Nankai University, Tianjin, China

**Keywords:** delayed retirement, subjective wellbeing, social capital, sense of achievement, time allocated to grandchild care

## Abstract

As China prepares to implement delayed retirement policies in response to population aging, understanding the impact of extended working lives on older adults' wellbeing has become increasingly important. While prior studies have focused on economic outcomes, less is known about the subjective consequences of working beyond traditional retirement age. This paper examines the effect of delayed retirement on the subjective wellbeing of older adults in China, with attention to underlying mechanisms and potential heterogeneity across gender and income groups. Using micro-level survey data from a period when delayed retirement was voluntary rather than mandatory, we identify the impact of retirement deferral on subjective wellbeing. We further explore three potential mechanisms and conduct subgroup analyses by gender and household income levels. We find that delayed retirement significantly increases subjective wellbeing among older adults. The improvement appears to result from enhanced social capital and a stronger sense of achievement, while reduced participation in intergenerational caregiving may partly offset the benefits. Gender heterogeneity analysis reveals that the positive effect is significant only for men, likely due to the additional domestic responsibilities faced by women. Similarly, income heterogeneity shows that benefits accrue primarily to higher-income individuals, whereas lower-income individuals experience no significant gains, possibly due to the involuntary nature of their extended work. These findings suggest that delayed retirement has complex effects on wellbeing, shaped by both social roles and economic constraints. Policy efforts should promote flexible and equitable retirement transitions, improve workplace support for older workers, and address the family-level tensions that may arise from extended employment.

## Introducition

**Figure 1:**
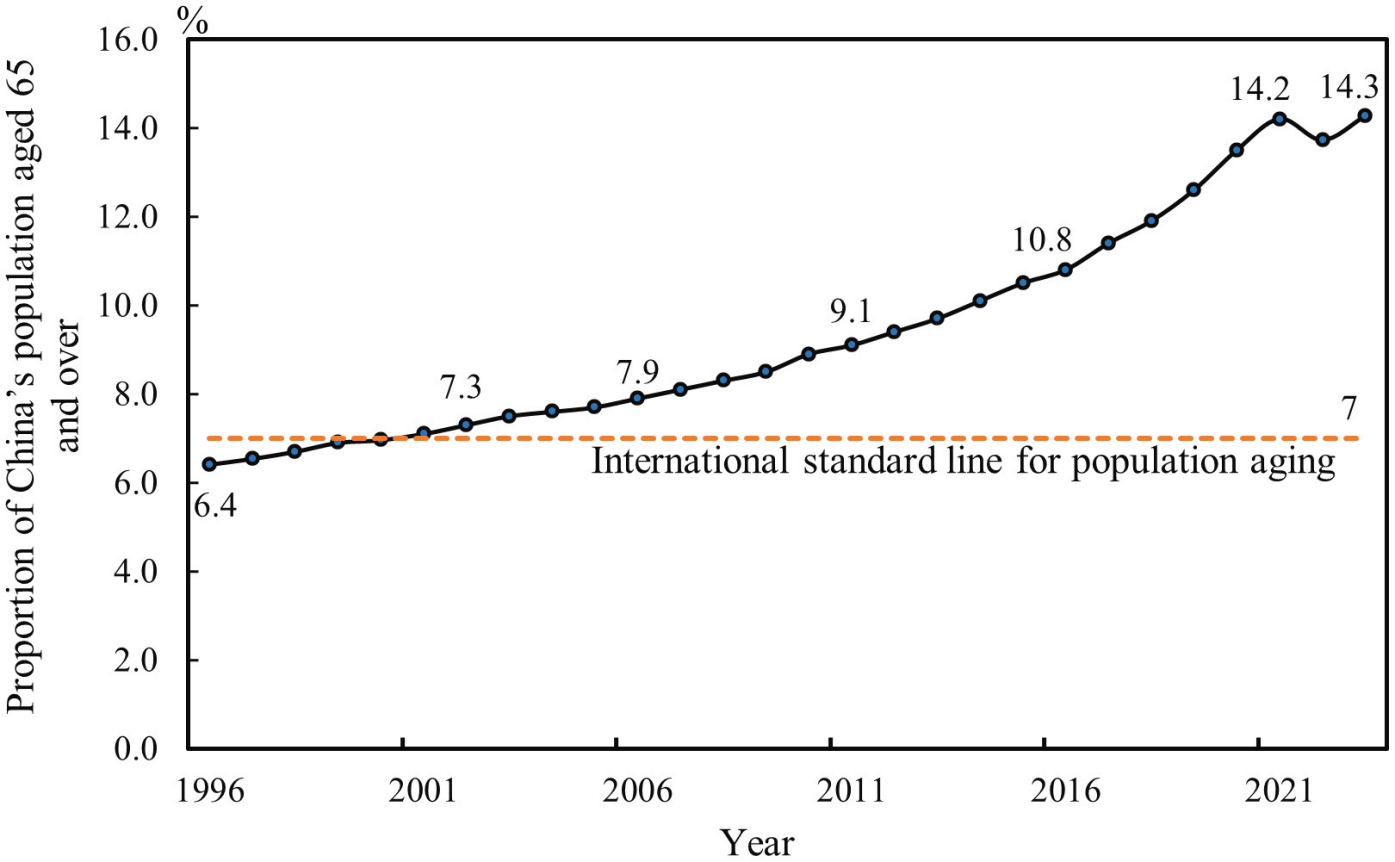
China's population aging trend.

China is experiencing a rapid demographic transition characterized by a sharp rise in the proportion of older individuals, driven in part by sustained improvements in healthcare and life expectancy. As shown in Figure 1, the share of the population aged 65 and above exceeded 7% in 2000, crossing the threshold for an aging society defined by the United Nations. This proportion has continued to rise at an accelerating pace, underscoring the deepening challenge of population aging.

Population aging is not only a demographic phenomenon but also a socioeconomic issue with profound implications for individual wellbeing. On the one hand, it poses growing pressure on public pension systems, healthcare infrastructure, and caregiving resources, potentially reducing the subjective wellbeing of older adults (1). On the other hand, it has prompted increasing policy attention toward improving the welfare of the older adult, including efforts to enhance their access to social participation and income security (2).

Retirement marks a critical life transition into old age, exerting a profound influence on individual wellbeing. On the one hand, it releases individuals from occupational stress and provides greater autonomy over their time, enabling increased engagement in family and social activities, which can enhance life satisfaction and psychological health (3). On the other hand, retirement may also lead to reduced income, weakened social roles, and disruptions to daily routines (4, 5), potentially diminishing one's sense of gain and social recognition (6). These changes may result in heightened feelings of loneliness and a loss of purpose (7), thereby exerting a negative impact on subjective wellbeing (8).

In response to the dual impact of retirement on wellbeing, some older adults choose to delay retirement, continuing to work in order to preserve their social roles and identity (9, 10), while also ensuring a stable source of income (11). As a key coping strategy during the transition into retirement, delayed retirement can help older individuals navigate early post-retirement adjustment challenges and foster a stronger sense of social participation and belonging (12).

As delayed retirement becomes increasingly common, the Chinese government introduced a major policy reform in September 2024 to address population aging, optimize labor resource allocation, and alleviate pension pressures. The Decision of the Standing Committee of the National People's Congress on the Implementation of a Gradual Increase in the Statutory Retirement Age outlines a phased approach to raising the statutory retirement age, with adjustments made concurrently for both male and female workers. Specifically, the retirement age for male employees will gradually increase from 60 to 63, while for female employees, the retirement age will rise from 50 and 55 to 55 and 58, respectively. The policy will be implemented progressively over a 15-year period and will officially take effect on January 1, 2025.

Nevertheless, the impact of delayed retirement on the subjective wellbeing of older adults remains uncertain. On the one hand, continued employment helps preserve social roles, reinforces a sense of self-worth, and enhances social engagement among older individuals (13, 14), thereby improving their life satisfaction and psychological wellbeing (4). Moreover, extending one's career allows older adults to maintain a stable income stream, which in turn alleviates potential post-retirement financial stress, sustains economic independence, and mitigates income-related anxiety (15, 16).

On the other hand, if delayed retirement conflicts with individual preferences or disrupts personal or family arrangements, it may generate adverse consequences. For instance, continued employment may reduce the time allocated to grandchild care, limiting older adults' ability to provide essential support to their children or grandchildren (17). This time constraint can increase physical strain (18) and introduce additional psychological stress (19), thereby exerting a negative impact on their overall wellbeing (20).

Building on the above analysis, this paper employs data from the China Family Panel Studies (CFPS) to empirically examine the impact of delayed retirement on the subjective wellbeing of older adults. Furthermore, it explores the multiple mechanisms through which delayed retirement influences wellbeing, particularly via enhanced sense of achievement and social capital. The findings not only provide empirical evidence to inform the refinement of delayed retirement policies, but also offer practical guidance for improving the quality of life and overall welfare of the older adult.

## Methods

### Data selection

The empirical data for this paper are sourced from the China Family Panel Studies (CFPS) database, which spans 25 provinces, municipalities, and autonomous regions and includes all household members within sampled families. The CFPS tracks data across individual, household, and community levels, covering a wide range of topics related to the economic and non-economic welfare of Chinese residents, including economic activity, social relationships, family dynamics, retirement status, and health. Given the lack of subjective wellbeing measures in the 2016 CFPS wave, the analysis is primarily based on data from the 2018, 2020, and 2022 waves. The 2014 wave is incorporated exclusively for robustness purposes. This data can be downloaded for free on http://www.isss.pku.edu.cn/cfps/.

The data processing procedure proceeds as follows. First, based on the statutory retirement ages in China−60 for men, 50 for female workers, and 55 for female cadres—we restrict the sample to individuals who have reached the official retirement age and have formally retired. Second, we identify delayed retirees according to the standard definition of delayed retirement. Third, we merge individual- and household-level characteristics of the older respondents. The final sample used for statistical analysis comprises 10,238 observations.

### Setting of the econometric model

This paper employs the following econometric model to identify the effects of delayed retirement policies on the subjective wellbeing of older adults:


(1) Wellbeingijt=α1+β1D_Retireijt+γ1Xijt+δj+δt+εijt,                        i=1,2,...,N

In this model, *Wellbeing*_*ijt*_ represents the subjective wellbeing of older adults individual in province in year *t*; *D*_Re*tire*_*ijt*_ captures the delayed retirement status of older individual *i* in province in year *t*; and β_1_ denotes the corresponding regression coefficient. *X*_*ijt*_ denotes a set of control variables, encompassing individual characteristics such as age, gender, marital status, years of education, health status, residence status alongside household characteristics such as net household income (adjusted to a 2010 base year) and family size. γ_1_ represents the coefficients associated with these control variables. δ_*j*_ captures province fixed effects, δ_*t*_ accounts for time fixed effects, and ε_*ijt*_ represents the random error term.

### Variable selection, definition, and descriptive statistics

#### Dependent Variable: subjective wellbeing

Subjective wellbeing is primarily a measure of individuals' emotional dispositions in daily life, with higher levels of positive emotions associated with greater wellbeing. In this paper, the subjective wellbeing indicator is based on responses to the survey question “How happy do you consider yourself to be?” Participants' scores, ranging from 1 to 10, are recoded as 1 for those with a score above 5 and 0 for those with a score of 5 or below.

#### Independent variable: delayed retirement

Since the delayed retirement policy has not yet been formally implemented, this paper defines delayed retirement behaviorally rather than institutionally, based on individuals' retirement timing relative to the statutory retirement age. The key explanatory variable is whether an individual has delayed retirement. Delayed retirement is defined as continuing to work after reaching the official retirement age (60 years for men, 50 years for female workers, and 55 years for female cadres) while still holding a labor contract. Conversely, individuals who have completed retirement formalities but cease work are classified as not having delayed retirement.

#### Mediating variables

The mediating variables in this paper are social capital, sense of achievement, and time allocated to grandchild care. Drawing on the work of Cai et al. (22), social capital is proxied by self-reported assessments of social relationships. Specifically, the survey asks respondents to rate the quality of their social relationships on a scale from 0 to 10, with higher scores indicating stronger social ties. To address potential endogeneity between sense of achievement and the explanatory variable, we construct a differenced measure of sense of achievement by subtracting the previous period's value, retaining only the instances where sense of achievement increases.

Following the definition by Zhu and Lv (23), sense of achievement refers to a subjective sense of benefit derived from objective achievements. We measure this using respondents' self-assessed social status in their local community, based on the survey item: “What is your social status in your local area?” Responses range from 1 (very low) to 5 (very high), with higher values indicating a higher perceived social status. We construct a binary variable equal to 1 if the reported status is 3, 4, or 5, and 0 otherwise.

Time allocated to grandchild care is defined using responses to the question: “Do you help your children with housework or childcare?” Respondents who answer affirmatively are classified as participating in intergenerational caregiving.

#### Instrumental variable

This paper uses formal employment status as an instrumental variable. The rationale is that formal employment directly affects the probability of delayed retirement but is plausibly exogenous to subjective wellbeing. Formal employment is defined based on respondents' answers to the question “Do you hold an established post?” Individuals employed in administrative agencies, public institutions, or state-owned enterprises are classified as formally employed, while all others are categorized as informally employed.

Control variables. Consistent with the existing literature, this paper includes two categories of control variables. The first captures individual characteristics: age, gender, marital status, years of education, health status, and residence status (urban vs. rural). The second captures household characteristics: net household income (adjusted to a 2010 base year) and family size.

Subjective wellbeing exhibits clear gender and life-cycle patterns: men report lower levels of wellbeing than women, and wellbeing tends to increase with age. Individuals who are married, more educated, or in better health report significantly higher levels of subjective wellbeing. Moreover, individuals with rural hukou tend to report lower levels of subjective wellbeing, likely due to differences in reference groups and psychological expectations (21). Household income and family size also play important roles in shaping the wellbeing of older adults.

Table 1 reports variable definitions and descriptive statistics. The mean value of subjective wellbeing among older adults is 0.819, suggesting a relatively high level of self-reported subjective wellbeing in this population. The mean of the delayed retirement indicator is 0.074, indicating that 7.4% of older adults in the sample chose to postpone retirement.

**Table 1:** Variable names and descriptive statistics.

**Variable**	**Variable name**	**Mean**	**Min**	**Max**
Dependent variable	Subjective wellbeing	0.819	0	1
Independent variable	Delayed retirement	0.074	0	1
Control variables	Age	69.75	55	99
	Gender	0.482	0	1
	Marital status	0.705	0	1
	Years of education	5.788	0	19
	Health status	0.21	0	1
	Residence status	0.599	0	1
	Family size	3.587	0	17
	Net household income (with 2010 as the base year)	10.067	0	16.247

Regarding demographic characteristics, the average age of respondents is 69.75 years, with a balanced gender distribution. About 70.5% are currently married, while the remaining 29.5% are unmarried, widowed, or divorced. The average years of education is 5.788, implying that most respondents did not complete junior high school—an outcome consistent with educational attainment levels for cohorts educated in the 1960s and 1970s. The mean value for health status is 0.21, indicating that the majority of older adults report being in relatively good health. Approximately 59.9% hold a rural Hukou. The average household size is 3.587 persons, and the mean of the log-transformed per capita household income is 9.772.

## Results

### Basic test

Column (1) of Table 2 presents the regression results without controlling for covariates or fixed effects. The coefficient on delayed retirement is positive and statistically significant at the 5 percent level, indicating that delayed retirement improves subjective wellbeing. Column (2) introduces individual-level controls, and the estimate remains stable in magnitude and significance. Column (3) further adds year and province fixed effects, yielding consistent results. We adopt Column (3) as our preferred specification, which suggests that delayed retirement increases the likelihood of reporting higher subjective wellbeing by ~3.3 percentage points.

**Table 2:** Effect of delayed retirement on older individuals' subjective wellbeing.

**Variable names**	**1**	**2**	**3**
	**Subjective wellbeing**	**Subjective wellbeing**	**Subjective wellbeing**
Delayed retirement	0.035^**^	0.034^**^	0.033^**^
	(0.014)	(0.016)	(0.016)
Age		0.004^***^	0.004^***^
		(0.001)	(0.001)
Gender		0.012	0.018^**^
		(0.010)	(0.009)
Marry		0.063^***^	0.063^***^
		(0.012)	(0.011)
Years of education		0.007	0.022
		(0.067)	(0.069)
Health status		−0.012	−0.015
		(0.010)	(0.010)
Residence status		−0.092^***^	−0.087^***^
		(0.009)	(0.010)
Family size		0.001	0.006^**^
		(0.002)	(0.002)
Net household income		0.001	0.002
		(0.001)	(0.002)
Time fixed effects	No	No	Yes
Province fixed effects	No	No	Yes
Constant	0.812^***^	0.540^***^	0.474^***^
	(0.005)	(0.057)	(0.055)
Observations	10,238	8,579	8,576

^**^p < 0.05; ^***^p < 0.01; Standard errors of the estimated coefficients are in parentheses, hereinafter. The standard errors in this paper are clustered at the province level.

The results on control variables reveal that older men report lower levels of subjective wellbeing compared to older women. Overall, subjective wellbeing increases with age and is significantly higher among married individuals and those with urban hukou. In addition, larger household size is positively associated with greater subjective wellbeing among the older adult.

### Robustness tests

#### Addressing endogeneity issues using instrumental variables

The baseline model may be subject to endogeneity concerns arising from omitted variable bias and other factors. Although the main regressions control for a rich set of individual and household characteristics, subjective wellbeing may still be influenced by unobserved factors such as personality traits or emotional disposition. Moreover, there is a potential concern of reverse causality: while delayed retirement may affect subjective wellbeing, it is also plausible that individuals with higher levels of wellbeing are more likely to postpone retirement. To address these issues, we use “formal employment status” as an instrumental variable for delayed retirement. We conduct a series of tests—including relevance, validity, and weak instrument diagnostics—to assess the suitability and strength of this instrument.

To address potential endogeneity in the relationship between delayed retirement and subjective wellbeing, we implement a two-stage least squares (2SLS) estimation using formal employment status as an instrumental variable. The estimation proceeds in two steps.

In the first stage, we examine the relevance of the instrument by regressing the delayed retirement on the instrument, formal employment status. As reported in Column (1) of Table 3, formal employment has a strong and statistically significant positive effect on delayed retirement, with the coefficient significant at the 1 percent level. This indicates that individuals in formal employment are more likely to postpone retirement, satisfying the relevance condition for a valid instrument.

**Table 3:** Instrumental variable method.

**Variable names**	**1**	**2**
	**First-stage**	**Second-stage**
	**Delayed retirement**	**Subjective wellbeing**
Delayed retirement		0.061^**^
		(0.031)
Formal employment status	0.065^***^	
	(0.003)	
Control variables	Yes	Yes
Time fixed effects	Yes	Yes
Province fixed effects	Yes	Yes
Constant	1.061^***^	0.493^***^
	(0.049)	(0.057)
Observations	8,652	8,652

The Anderson canonical correlation LM statistic is 679.342, strongly rejecting the null hypothesis of underidentification and confirming the model is identified. The Cragg-Donald Wald F statistic is 3732.119, which far exceeds the Stock-Yogo critical value of 16.38 at the 10 percent significance level. This provides strong evidence against the null hypothesis of weak instruments. The Hansen J statistic equals zero in all specifications, indicating that the overidentifying restrictions are satisfied. Collectively, these tests confirm that formal employment status is a valid and strong instrument. ^**^p < 0.05; ^***^p < 0.01; Standard errors of the estimated coefficients are in parentheses, hereinafter.

In the second stage, we replace the endogenous regressor with its fitted values from the first stage and regress subjective wellbeing on these predicted values. The results, shown in Column (2) of Table 3, reveal that the fitted values are positively and significantly associated with subjective wellbeing at the 5 percent level. The coefficient estimate is consistent in sign with the baseline OLS results, suggesting that the positive effect of delayed retirement on subjective wellbeing remains robust even after accounting for potential endogeneity.

#### Lagging the independent variable by one period

To examine the model's sensitivity to temporal dynamics and address potential endogeneity concerns, we re-estimate the specification using a one-period lag of the key explanatory variable. As shown in Column (1) of Table 4, the lagged variable remains positively signed and statistically significant at the 5 percent level. This result offers additional evidence in support of the robustness of the baseline estimates.

**Table 4:** Robustness tests results.

**Variable names**	**1**	**2**	**3**	**4**
Delayed retirement		0.026^**^	0.084^**^	0.028^**^
		(0.015)	(0.039)	(0.014)
L. Delayed retirement	0.056^**^			
	(0.027)			
Control variables	Yes	Yes	Yes	Yes
Time fixed effects	Yes	Yes	Yes	Yes
Province fixed effects	Yes	Yes	Yes	Yes
Constant	0.153	0.525^***^		0.490^***^
	(0.170)	(0.358)		(0.047)
Observations	3,023	8,576	8,576	10,765

^**^p < 0.05; ^***^p < 0.01; Standard errors of the estimated coefficients are in parentheses, hereinafter. Columns 2 and 3 report average marginal effects rather than raw regression coefficients.

#### Modification of model specification

Since the dependent variable—subjective wellbeing—is binary, we estimate a Probit model as a robustness check in lieu of the fixed effects specification. Column (2) of Table 4 presents the results. The coefficient on delayed retirement remains positive and statistically significant at the 5 percent level, indicating that the estimated effect is robust to alternative model specifications. These results strengthen the credibility of the baseline findings and underscore the consistency of the positive relationship between delayed retirement and subjective wellbeing.

#### Modifying the definition of the dependent variable

As an additional robustness check, we recode the dependent variable as an ordered outcome using responses to the survey question “How happy do you feel?”, scored on a scale from 1 to 10. We then estimate an ordered Probit model as an alternative to the fixed effects specification. Column (3) of Table 5 reports the results. The coefficient on delayed retirement remains positive and statistically significant at the 5 percent level. These findings confirm that the positive relationship between delayed retirement and subjective wellbeing is robust to alternative codings of the dependent variable and aligns closely with our baseline estimates.

**Table 5:** The VIFs for all covariates.

**Variable**	**VIF**
Delayed retirement	1.14
Age	1.30
Gender	1.12
Marital status	1.12
Years of education	1.01
Health status	1.01
Residence status	1.06
Family size	1.16
Net household income (with 2010 as the base year)	1.17
Mean VIF	1.12

#### Expanding the sample to include data from 2014

Due to the absence of instrumental variable data in 2014, the baseline regressions exclude observations from that wave. As an additional robustness check, we re-estimate the model using pooled data from the 2014, 2018, 2020, and 2022 survey waves. The results, reported in Column (4) of Table 5, show that the coefficient on delayed retirement remains positive and statistically significant at the 5 percent level. This suggests that the estimated effect is robust to the inclusion of additional survey waves and continues to support the main conclusion that delayed retirement enhances subjective wellbeing.

#### Issues of multicollinearity

To mitigate concerns about multicollinearity, we calculate the Variance Inflation Factors (VIFs) for all covariates. The literature generally considers VIF values below 10 as indicative of negligible multicollinearity. As reported in Table 5, the average VIF is 1.12, with the maximum value being 1.3, suggesting that multicollinearity is unlikely to bias our estimates.

### Mechanism analysis

This section employs a stepwise regression approach to examine the potential mechanisms through which delayed retirement affects individuals' subjective wellbeing, focusing on three key channels: social capital, sense of achievement, and time allocated to grandchild care. Column (1) of Table 6 presents the baseline regression, which replicates the main finding that delayed retirement significantly improves subjective wellbeing, without accounting for the mediating variables. Columns (2), (4), and (6) of Table 6 then report the effects of delayed retirement on the proposed mechanisms. The results indicate that delayed retirement significantly enhances both social capital and sense of achievement, while it significantly reduces time allocated to grandchild care.

**Table 6:** Results of the mechanism analysis.

**Variable names**	**1**	**2**	**3**	**4**	**5**	**6**	**7**
	**Subjective wellbeing**	**Social capital**	**Subjective wellbeing**	**Sense of achievement**	**Subjective wellbeing**	**Time allocated to grandchild care**	**Subjective wellbeing**
Delayed retirement	0.033^**^	0.246^***^	0.027^*^	0.074^*^	0.029^*^	−0.047^**^	0.026^*^
	(0.016)	(0.083)	(0.014)	(0.044)	(0.016)	(0.019)	(0.014)
Social capital			0.071^***^				
			(0.002)				
Sense of achievement					0.059^***^		
					(0.004)		
Time allocated to grandchild care							0.018^**^
							(0.008)
Control variables	Yes	Yes	Yes	Yes	Yes	Yes	Yes
Time fixed effects	Yes	Yes	Yes	Yes	Yes	Yes	Yes
Province fixed effects	Yes	Yes	Yes	Yes	Yes	Yes	Yes
Constant	0.474^***^	5.857^***^	0.014	1.714^***^	0.373^***^	0.460^***^	0.790^***^
	(0.055)	(0.286)	(0.051)	(0.153)	(0.055)	(0.065)	(0.014)
Observations	8,576	8,576	8,576	8,576	8,576	8,576	10,161

^*^p < 0.1; ^**^p < 0.05; ^***^p < 0.01; Standard errors of the estimated coefficients are in parentheses, hereinafter.

Column (3) of Table 6 presents the results after controlling for social capital in the baseline regression of delayed retirement on subjective wellbeing. The coefficient on delayed retirement decreases in magnitude and significance, while social capital itself exhibits a significantly positive effect on subjective wellbeing. These findings suggest that social capital partially mediates the relationship between delayed retirement and subjective wellbeing. In particular, continued labor force participation enhances older adults' social capital, which in turn contributes to greater subjective wellbeing.

Column (5) of Table 6 reports the regression results after controlling for sense of achievement. The coefficient on delayed retirement decreases in both magnitude and statistical significance, while perceived sense of achievement a significantly positive association with subjective wellbeing. These findings provide evidence that sense of achievement serves as a partial mediator in the relationship between delayed retirement and subjective wellbeing. In other words, delayed retirement enhances older adults' sense of achievement, which in turn contributes to improved subjective wellbeing.

Column (7) of Table 6 presents the results after controlling for time allocated to grandchild care. The time allocated to grandchild care is significantly positive, indicating that providing time-intensive caregiving improves subjective wellbeing among older adults. Meanwhile, the coefficient on delayed retirement declines substantially in both magnitude and significance, suggesting that time allocated to grandchild care is another important channel through which delayed retirement affects subjective wellbeing. Specifically, delayed retirement reduces the time allocated to grandchild care, which in turn diminishes one pathway through which older adults derive wellbeing—thereby generating a negative indirect effect.

Taken together, although delayed retirement exerts a negative effect on subjective wellbeing by reducing time allocated to grandchild care, the magnitude of this effect is not sufficient to offset the positive impacts arising from increased social capital and enhanced sense of achievement. As a result, the net effect of delayed retirement remains positive, ultimately leading to an overall improvement in subjective wellbeing.

### Heterogeneity analysis

Previous research has shown that delayed retirement can positively influence subjective wellbeing by enhancing perceived gain and social capital, while it may also exert a negative effect by reducing time spent on intergenerational caregiving. In the specific cultural context of China, older women are typically the main providers of intergenerational care within the family, whereas older adults with higher household income are more inclined to pursue perceived gain and social capital. Therefore, this paper further conducts heterogeneity analysis by gender and household income, which also serves to test the robustness of the underlying mechanisms.

#### Gender heterogeneity

While delayed retirement generally enhances the subjective wellbeing of older adults, its effects vary by gender due to differences in the roles and responsibilities that men and women typically assume within the family. As shown in Columns (1) and (2) of Table 7, the gender-specific regression results indicate that delayed retirement significantly increases the subjective wellbeing of male older adults. In contrast, although the estimated effect for female older adults is also positive, it does not reach statistical significance.

**Table 7:** Results of the heterogeneity analysis.

**Variable names**	**1**	**2**	**3**	**4**
	**Gender**		**Income**	
	**Older women**	**Older men**	**Low**	**High**
Delayed retirement	0.048 (0.029)	0.031^*^ (0.017)	0.014 (0.019)	0.045^*^ (0.027)
Control variables	Yes	Yes	Yes	Yes
Time fixed effects	Yes	Yes	Yes	Yes
Province fixed effects	Yes	Yes	Yes	Yes
Constant	0.383^***^ (0.070)	0.649^***^ (0.085)	0.613^***^ (0.071)	0.382^***^ (0.074)
Observations	4,404	4,211	3,609	4,967

^*^p < 0.1; ^***^p < 0.01; Standard errors of the estimated coefficients are in parentheses, hereinafter.

#### Household income heterogeneity

Household income plays a critical role in shaping subjective wellbeing. Older individuals in high-income families have greater access to disposable resources, allowing them to substitute their time in intergenerational caregiving with market-based childcare services such as private nurseries or live-in nannies. Moreover, higher-income individuals are more likely to prioritize intangible outcomes—such as social capital and a sense of achievement—thereby enhancing their wellbeing through these channels. In contrast, older adults in low-income households often engage in low-skilled occupations with limited flexibility to balance work and family responsibilities. As a result, these families rely heavily on unpaid grandparental care, and lack the financial capacity to pursue market-based alternatives. Consequently, the negative effect of reduced time allocated to grandchild care due to delayed retirement is substantially amplified in these households, likely offsetting the positive effects of increased social capital and sense of achievement.

To examine whether the effect of delayed retirement on subjective wellbeing varies by household income, we divide the sample into high-income and low-income groups based on the median level of net household income. The subgroup regression results are presented in Columns (3) and (4) of Table 7. We find that the positive effect of delayed retirement on subjective wellbeing is more pronounced and statistically significant among older individuals in high-income households, whereas the effect is not statistically significant among those in low-income households.

## Discussion

This paper investigates the impact of delayed retirement on the subjective wellbeing of older adults. During the sample period, China had not yet implemented a mandatory delayed retirement policy; hence, this paper focuses on the effects of voluntary delayed retirement behavior. The empirical findings suggest that delayed retirement significantly enhances the subjective wellbeing of older adults, consistent with the conclusions of Gu et al. (3). We argue that this effect reflects the interplay of three mechanisms: a positive effect through the enhancement of social capital and an increased sense of achievement, and a negative effect arising from the reduction in time allocated to grandchild care within the household. While the latter exerts a downward pressure on wellbeing, the net effect of delayed retirement remains positive, highlighting the predominance of the psychological and social benefits associated with continued labor force participation.

On the one hand, delayed retirement enables older adults to remain engaged in the workforce and maintain close ties with society, thereby fostering the accumulation and enhancement of social capital (10). Rich social capital provides emotional, informational, and instrumental support, which helps mitigate common psychological challenges among the older adult—such as loneliness, helplessness, and marginalization (7). It also promotes healthier behavioral patterns and psychological states (12), ultimately contributing to an improvement in their subjective wellbeing.

On the other hand, delayed retirement can also enhance older adults' subjective wellbeing by strengthening their sense of achievement. The “s sense of achievement” is a subjective perception that reflects individuals' experiential satisfaction regarding what they have attained in terms of material resources, social status, and personal value (23). By extending the period during which older individuals receive wages, delayed retirement improves their material sense of achievement, providing them with greater economic security. In addition, it reinforces the perception of being “still needed” and “still contributing to society,” thereby enhancing their sense of personal value. Continued labor market participation also affords older adults sustained respect and recognition from colleagues, employers, and the broader community, which contributes to a stronger sense of social identity. As a key psychological foundation of subjective wellbeing (9), this multi-dimensional sense of achievement plays a significant role in promoting happiness in later life.

However, delayed retirement may also undermine older adults' subjective wellbeing by reducing their capacity to engage in intergenerational caregiving. For those who bear caregiving responsibilities for grandchildren, continued employment introduces role and time conflicts that hinder their ability to fulfill such duties (17). This tension is particularly salient in dual-earner households, where older adults often serve as primary caregivers for their grandchildren. In these contexts, active involvement in grandchildren's upbringing serves as a meaningful source of emotional fulfillment and life satisfaction. By reducing the time available for intergenerational interactions, delayed retirement can weaken familial bonds and diminish opportunities for emotional connection, potentially leading to feelings of loss, loneliness, and psychological distress (19). Consequently, the disruption of caregiving roles may exert a negative influence on older adults' overall happiness.

This paper further explores heterogeneity in the effect of delayed retirement on subjective wellbeing across gender and income groups. The gender-specific results suggest that delayed retirement significantly enhances the subjective wellbeing of older men, but the effect is statistically insignificant for older women. This gender gap may be attributed to traditional gender role expectations, under which women often bear dual responsibilities in both the labor market and the household. In addition to employment, women are typically expected to engage in domestic chores, caregiving, and intergenerational childcare. Delayed retirement may thus intensify role conflicts and exacerbate time and psychological stress for older women (19), ultimately undermining their wellbeing. Moreover, implicit bias and age-related discrimination in the workplace may prevent older female workers from experiencing the same sense of accomplishment or self-worth associated with continued employment (20). These factors collectively contribute to the lack of significant improvement in subjective wellbeing among older women who delay retirement.

The results on income heterogeneity reveal that the positive effect of delayed retirement on subjective wellbeing is significant only among older adults in high-income households, while the effect is statistically insignificant for those in low-income households. A plausible explanation is that, for low-income individuals, the decision to delay retirement is often driven by financial necessity rather than personal preference or intrinsic motivation. Being “forced to work” under economic pressure may induce negative emotions, fatigue, and a sense of powerlessness, thereby offsetting any potential gains in wellbeing. In contrast, higher-income individuals are more likely to pursue greater social capital and a stronger sense of fulfillment. For them, the marginal benefit of continued employment—in terms of income and identity reinforcement—is relatively high, while the opportunity cost, such as reduced time for family interaction or intergenerational caregiving, is comparatively lower. As a result, delayed retirement has a more pronounced positive impact on their subjective wellbeing.

Based on the empirical findings, this study offers three policy recommendations. First, a differentiated and flexible approach to delayed retirement policy should be promoted, reflecting principles of equity and adaptability. Policymakers should take into account individual differences such as gender, occupation type, health status, and economic condition, and adopt a gradual and flexible strategy tailored to local and individual circumstances.

Second, institutional support for older adults who choose to remain in the workforce should be improved to enhance their sense of gain and social recognition. Delayed retirement should not merely be about extending years of employment, but rather about promoting older adults' professional value and subjective wellbeing. Efforts should focus on strengthening the policy framework and platform for senior employment, facilitating the accumulation of social capital, and offering older workers greater respect, participation, and opportunities for advancement—thereby reinforcing their work identity and sense of accomplishment.

Third, greater attention should be paid to balancing intergenerational family relationships and mitigating caregiving conflicts arising from delayed retirement. As older adults spend more time at work, their availability for intergenerational caregiving may decline, potentially reducing family satisfaction and subjective wellbeing. To address this, a family-supportive policy framework should be established by expanding accessible and affordable childcare services, enhancing the coverage and quality of public early education, and improving the implementation and structure of parental leave policies. These measures can help ease the caregiving burden on older adults and resolve time allocation conflicts across generations, ultimately achieving a more effective coordination between work responsibilities and family obligations.

## Data Availability

The original contributions presented in the study are publicly available. This data can be found here: https://www.isss.pku.edu.cn/cfps/en/.
